# Involvement of hepatocyte growth factor in increased integrin expression on HepG2 cells triggered by adhesion to endothelial cells.

**DOI:** 10.1038/bjc.1997.8

**Published:** 1997

**Authors:** N. Kawakami-Kimura, T. Narita, K. Ohmori, T. Yoneda, K. Matsumoto, T. Nakamura, R. Kannagi

**Affiliations:** Program of Experimental Pathology, Research Institute, Aichi Cancer Center, Nagoya, Japan.

## Abstract

Adhesion of cancer cells to vascular endothelium is an important step in haematogenous metastasis of cancer. A human hepatocellular carcinoma cell line, HepG2, strongly adheres to human umbilical vein endothelial cells (HUVECs) through the interaction of E-selectin and its carbohydrate ligand sialyl Lewis X. In this study, we investigated alteration in integrin expression on HepG2 cells, which follows the selectin-mediated initial adhesion of HepG2 cells to HUVECs. Expression of alpha2beta1 integrin was markedly increased when the HepG2 cells adhered to HUVECs. Among the tested cytokines that are known to be produced by endothelial cells, recombinant hepatocyte growth factor (rHGF) could replace the effect of HUVECs, and a similar increase in integrin expression was observed by the addition of 20 ng ml-1 rHGF to HepG2. The increment of alpha2beta1 integrin expression was significantly inhibited by anti-HGF neutralizing antibody treatment. HepG2 cells expressed alpha2, alpha6, beta1, and beta4 integrin subunits, but expression of integrins other than alpha2beta1 was not affected by the rHGF treatment. The rHGF treatment of HepG2 cells resulted in augmented adhesion to immobilized collagen. This augmentation in adhesion to collagen was completely blocked by the addition of anti-alpha2- or anti-beta1-integrin antibody. In double-chamber chemoinvasion experiments, transmigration of the HepG2 cells through extracellular matrix (ECM) gel was significantly accelerated by co-cultivation with HUVECs. A similar level of enhancement in transmigration activity of the cancer cells was observed by the addition of rHGF. Our interpretation of the results described above is that the cancer cells received stimulation from cytokines, such as HGF, presented by vascular endothelial cells, following the initial adhesion of cancer cells via selectins. This resulted in the secondary increment in the expression of cell adhesion molecules, such as the alpha2beta1 integrin, and led to the augmented adhesive activities of cancer cells towards extracellular matrices at vascular walls. We suggest that this sequence of events is involved in the facilitated migration of some cancer cells to extravascular tissues.


British Journal of Cancer (1997) 75(1), 47–53
© 1997 Cancer Research Campaign

### Involvement of hepatocyte growth factor in increased integrin expression on HepG2 cells triggered by adhesion to endothelial cells

**N Kawakami-Kimura[1], T Narita[1], K Ohmori[2], T Yoneda[2], K Matsumoto[3], T Nakamura[3] and R Kannagi[1,4]**

[1]Program of Experimental Pathology, Research Institute, Aichi Cancer Center, Nagoya, 464; [2]Department of Laboratory Medicine, Kyoto University, School of Medicine, Kyoto, 606; [3]Department of Biochemical Oncology, Osaka University, School of Medicine, Osaka, 565, Japan

**Summary** Adhesion of cancer cells to vascular endothelium is an important step in haematogenous metastasis of cancer. A human hepatocellular carcinoma cell line, HepG2, strongly adheres to human umbilical vein endothelial cells (HUVECs) through the interaction of E-selectin and its carbohydrate ligand sialyl Lewis X. In this study, we investigated alteration in integrin expression on HepG2 cells, which follows the selectin-mediated initial adhesion of HepG2 cells to HUVECs. Expression of $\alpha_2\beta_1$ integrin was markedly increased when the HepG2 cells adhered to HUVECs. Among the tested cytokines that are known to be produced by endothelial cells, recombinant hepatocyte growth factor (rHGF) could replace the effect of HUVECs, and a similar increase in integrin expression was observed by the addition of 20 ng $ml^{-1}$ rHGF to HepG2. The increment of $\alpha_2\beta_1$ integrin expression was significantly inhibited by anti-HGF neutralizing antibody treatment. HepG2 cells expressed $\alpha_2$, $\alpha_6$, $\beta_1$ and $\beta_4$ integrin subunits, but expression of integrins other than $\alpha_2\beta_1$ was not affected by the rHGF treatment. The rHGF treatment of HepG2 cells resulted in augmented adhesion to immobilized collagen. This augmentation in adhesion to collagen was completely blocked by the addition of anti-$\alpha_2$- or anti-$\beta_1$-integrin antibody. In double-chamber chemoinvasion experiments, transmigration of the HepG2 cells through extracellular matrix (ECM) gel was significantly accelerated by co-cultivation with HUVECs. A similar level of enhancement in transmigration activity of the cancer cells was observed by the addition of rHGF. Our interpretation of the results described above is that the cancer cells received stimulation from cytokines, such as HGF, presented by vascular endothelial cells, following the initial adhesion of cancer cells via selectins. This resulted in the secondary increment in the expression of cell adhesion molecules, such as the $\alpha_2\beta_1$ integrin, and led to the augmented adhesive activities of cancer cells towards extracellular matrices at vascular walls. We suggest that this sequence of events is involved in the facilitated migration of some cancer cells to extravascular tissues.

**Keywords:** cancer metastasis; cell adhesion; selectin; sialyl Lewis X; integrins; hepatocyte growth factor

Haematogenous metastasis of cancer is a complicated process consisting of multiple steps. The attachment of cancer cells to vascular endothelium is possibly initiated by the cell adhesion mediated by E-selectin on endothelial cells and carbohydrate ligands on cancer cells (Hakomori, 1992; Majuri et al, 1992; Dejana et al, 1992; Takada et al, 1991, 1993). The only exceptions known are non-epithelial malignant cells, such as melanoma or neuroblastoma and fibrosarcoma, which bind to endothelial cells mainly through VCAM-1. The carbohydrate determinants, sialyl Lewis A and sialyl Lewis X, expressed on cancer cells serve as ligands for E-selectin (Lowe et al, 1990; Phillips et al, 1990; Takada et al, 1991, 1993). Sialyl Lewis A is primarily involved in the adhesion of cancers of digestive organs, while sialyl Lewis X is mainly involved in the adhesion of liver, breast, lung and ovary cancer cells to endothelial cells (Majuri et al, 1992; Dejana et al, 1992; Takada et al, 1991, 1993).

The selectin–carbohydrate interaction can be regarded as an important factor that facilitates adhesion of cancer cells to endothelial cells during the course of haematogenous metastasis (Merwin et al, 1992; Giavazzi et al, 1993). In vitro experiments indicated that E-selectin expression on endothelial cells is induced by cytokines, such as interleukin 1 (IL-1) and/or tumour necrosis factor alpha (TNFα) (Bevilacqua et al, 1987), suggesting that this cell adhesion system is heavily involved, especially in cancer metastasis into damaged and/or inflamed tissues. Some cancer cells are known to express IL-1 and other cytokines that activate endothelial cells (Li et al, 1992; Alexandroff et al, 1994; Hayashi et al, 1994), and these cytokines, through the induction of cell surface E-selectin, would also facilitate the adhesion of cancer cells to endothelial cells. Serum E-selectin levels are known to be elevated in patients with cancers, reflecting the enhanced expression of E-selectin in the vessel walls of these patients (Banks et al, 1993; Ye et al, 1995). Recent immunohistochemical studies also indicate that small vessels adjacent to cancer nests express E-selectin strongly (Ye et al, 1995). All these findings suggest the importance of E-selectin-mediated cell adhesion in cancer metastasis.

However, the sequence of events following the initial step of cell adhesion and leading to the extravasation of cancer cells remains largely unknown. Here, we have studied the expression of integrins on cancer cells after the cells underwent the selectin-mediated initial adhesion process to endothelial cells. We also tried to identify the molecular species of affected integrins and cytokines involved in the regulation of their expression.

*Received 14 May 1996*
*Revised 30 July 1996*
*Accepted 31 July 1996*

*Correspondence to:* R Kannagi, Laboratory of Experimental Pathology, Research Institute, Aichi Cancer Center, 1-1 Kanokoden, Chikusaku, Nagoya, 464, Japan

### MATERIALS AND METHODS

### Cell culture, chemical reagents and antibodies

The human hepatocellular cancer cell line, HepG2 (ATCC, Rockville, MD, USA), was maintained in Dulbecco's modified Eagle medium (DMEM, GIBCO/BRL, Grand Island, NY, USA), supplemented with 10% fetal calf serum (FCS). Human umbilical vein endothelial cells (HUVECs, Seikagaku Kogyo, Tokyo, Japan) were maintained in Daigo's T medium (Nissui Seiyaku, Tokyo) supplemented with 10% FCS and 2 ng ml$^{-1}$ recombinant bFGF (kindly provided by Takeda Pharmaceutical Osaka, Japan). Purity of HUVECs was ascertained by flow cytometric analysis using anti-factor VIII antibody and was more than 99% throughout the experiments described in this study.

Recombinant human IL-1β was kindly provided by Otsuka Pharmaceutical (Tokushima, Japan). Human MIP-1β, recombinant human IL-8 and human keratinocyte growth factor (KGF) were obtained from Pepro Tech (Rocky Hill, NJ, USA). Recombinant human hepatocyte growth factor (rHGF) was purified from culture medium of CHO cells transfected with plasmid containing the human HGF cDNA (Nakamura et al, 1989). Recombinant human heparin-binding epidermal growth factor (rHB-EGF) was kindly provided by Dr S Higashiyama, Osaka University.

Antibodies directed to CD11a ($\alpha_l$-subunit, MHM24) and CD18 ($\beta_2$-subunit, MHM23) integrins were purchased from Dako (Glostrup, Denmark); those directed to CD11b ($\alpha_m$-subunit, D12), CD11c ($\alpha_x$-subunit, S-HCL-3), $\beta_3$ (7F12) and $\beta_4$ (AA3) subunits were from Becton Dickinson (Mountain View, CA, USA); anti-CD29 ($\beta_1$-subunit, 4B4) was from Coulter Immunology (Hialeah, FL, USA); anti-CD49a antibody ($\alpha_1$-subunit, TS2/7) was from T Cell Diagnostics (Cambridge, MA, USA); anti-CD49e antibody ($\alpha_5$-subunit, KH33) was from Seikagaku Kogyo (Tokyo, Japan); and antibodies to CD49b ($\alpha_2$subunit, Gi9), CD49c ($\alpha_3$-subunit, M-KID2), CD49d ($\alpha_4$-subunit, HP2/1) and CD49f ($\alpha_6$-subunit, GoH3) were obtained from Immunotech (Marseille, France).

### Monolayer cell adhesion assay using HUVECs

HUVECs were stimulated with 2 ng ml$^{-1}$ recombinant IL-1β for 4 h in 24-well plates. HepG2 cells ($5 \times 10^5$ cells per well) were added and the plate was incubated with rotation at 90 r.p.m. for 20 min at room temperature (Takada et al, 1991, 1993). After non-adherent cells were washed out three times with phosphate-buffered saline (PBS), the number of attached cells was counted directly under a microscope. Monoclonal anti-E-selectin, anti-ICAM-1 and anti-VCAM-1 antibodies (BBA2, BBA4 and BBA6, all murine IgG$_1$) were obtained from British Biotechnology, Abingdon, Oxon, UK. These antibodies were preincubated with HUVECs at 50 μg ml$^{-1}$ for 30 min at 37°C before the adhesion experiments with HepG2 cells for inhibition. Monoclonal antibodies SNH-3 (specific to sialyl Lewis X, kindly supplied by Dr Sen-Itiroh Hakomori, Biomembrane Institute, Seattle, WA, USA) and 2D3 (specific to sialyl Lewis A, established in our laboratory) were preincubated with HepG2 cells at 25 μg ml$^{-1}$ for 30 min at room temperature before application to the monolayer of HUVECs (Takada et al, 1991, 1993).

### Flow cytometric analysis for cell surface integrin expression

To study the change in integrin expression induced by selectin-mediated cell adhesion, HepG2 cells were adhered to the monolayer of HUVECs and co-cultured for 24 h at 37°C. To assess the effect of cytokines on integrin expression, HepG2 cells were cultured with 20 ng ml$^{-1}$ rHGF or other cytokines for 24 h at 37°C. Flow cytometric analysis of HepG2 cells was performed using FACScan (Becton Dickinson Immuno-cytometry System, Mountain View, CA, USA) as described previously (Ohmori et al, 1993). The indirect immunofluorescence method was applied for staining of HepG2 cell integrins, using an anti-integrin antibody as the first antibody and a fluorescein isothiocyanate (FITC)-labelled mouse anti-Ig as the second antibody (Cappel, Malvern, PA, USA).

Experiments for the inhibition of integrin expression were performed using rabbit antisera raised against human rHGF (Montesano et al, 1991). Rabbit anti-IL-1β (kindly supplied by Otsuka Pharmaceutical, Tokushima, Japan) and anti-bFGF antibodies (kindly provided by Takeda Pharmaceutical, Osaka, Japan) served as control antibodies in these experiments. These antibodies were added at 1/50 – 1/250 dilution to the mixed culture of HepG2 and HUVECs for 24 h at 37°C before flow cytometric analysis.

### Cellular enzyme-linked immunosorbent assay (CELISA) for cell surface integrins

Quantitative CELISA for total integrin expression was performed on the monolayer of HepG2 cells that were grown in 96-well plates. After incubation with culture medium containing 20 ng ml$^{-1}$ rHGF for 24 h unless otherwise indicated, the plate was washed twice with PBS and fixed by adding 0.1% glutaraldehyde for 1 h at room temperature. The plate was washed three times with PBS and unbound surfaces were blocked with 5% bovine serum albumin (BSA) in PBS overnight at 4°C. After washing twice with PBS, 50 μl of the primary antibodies was added to the wells and incubated for 1 h at room temperature. After washing three times with PBS, a 1:100 dilution of peroxidase-conjugated goat anti-mouse IgG (Zymed Laboratories, San Francisco, CA, USA) was added as the secondary antibody and incubated for 1 h at room temperature. The excess enzyme conjugates were then removed by washing three times with PBS, and 50 μl of the substrate, *O*-phenylenediamine in citrate buffer, containing 0.015% hydrogen peroxide, was added to each well for 10–15 min at room temperature. The colour reaction was stopped with 2M sulphuric acid (50 μl per well), and absorbance was measured using a microplate reader (Tosoh, Tokyo) at 492 nm.

### Cell attachment assay to ECM molecules

The 24-well plates were coated with 5 μg ml$^{-1}$ collagen I (Seikagaku Kogo, Tokyo) or 20 μg ml$^{-1}$ laminin (Takara Shuzo, Otsu, Japan) at 4°C overnight (Staatz et al, 1989; Carter et al, 1990), and the wells were washed three times with PBS. Unbound surfaces were blocked with 0.5% BSA in PBS for 1 h, and again the wells were washed three times with PBS. HepG2 cells ($5 \times 10^5$), after the preincubation with 20 ng ml$^{-1}$ rHGF for 24 h, were added in a volume of 500 μl per well to each substrate-coated well and then incubated for 30 min at 37°C. The wells were then washed three times with PBS to remove unattached cells. The number of attached cells was counted directly under a microscope.

### Chemoinvasion assay

Invasion chambers with 6-mm-diameter filters (8-μm pore size) were coated with 50 μg ml$^{-1}$ Matrigel (Collaborative Biomedical Products, Bedford, MA, USA). The coated chambers were placed

 © Cancer Research Campaign 1997

**Figure 1** Contribution of known adhesion molecules and their ligands to the adhesion of HepG2 cells to rIL-1β-activated HUVECs. (**A**) Results of pretreatment of HUVECs with anti-E-selectin, anti-ICAM-1 or anti-VCAM-1 antibody. HUVECs were treated with the respective antibody (50 µg ml$^{-1}$) for 30 min before the adhesion experiment. (**B**) Results of pretreatment of HepG2 cells with anti-sialyl Lewis A (2D3), anti-sialyl Lewis X (SNH-3) or a mixture of both antibodies. HepG2 cells were treated with antibodies (25 µg ml$^{-1}$) for 30 min before the adhesion experiment.

**Figure 2** Flow cytometric analysis of augmentation of $\alpha_2\beta_1$ integrin expression on HepG2 cells by the co-cultivation with HUVECs (**A**) or by the addition of rHGF (**B**). In **A**, HepG2 cells were co-cultured with (—) or without (—) HUVECs for 24 h. In **B**, HepG2 cells were cultured in DMEM in the presence (—) or absence (—) of 20 ng ml$^{-1}$ rHGF for 24 h. Stained using anti-CD49b antibody

**Figure 3** Effects of various cytokines on $\alpha_2\beta_1$ integrin expression on HepG2 cells. HepG2 cells were cultured in DMEM containing rHGF (20 ng ml$^{-1}$), KGF (100 ng ml$^{-1}$), IL-1β (2 ng ml$^{-1}$), bFGF (2 ng ml$^{-1}$), HB-EGF (10 ng ml$^{-1}$), TGF-β (5 ng ml$^{-1}$), IL-8 (10 ng ml$^{-1}$) or MIP-1β (100 ng ml$^{-1}$) for 24 h, and expression of the $\alpha_2\beta_1$ integrin was assessed by CELISA using anti-CD49b antibody. The results are expressed as percentage absorbance of non-treated HepG2 cells. Bars show s.d.

in 24-well plates. To examine the effect of adhesion to HUVECs on the transmigration activity, HUVEC monolayers were formed in the chambers, and HepG2 cells (7 × 10$^4$) suspended in DMEM were added to the chambers. For examination of the effect of HGF, HepG2 cells were added to the upper chambers with 20 ng ml$^{-1}$ rHGF. The medium containing 20 µg ml$^{-1}$ collagen I or medium alone was added to each lower well of the 24-well plate. Cells were cultured at 37°C for 72 h. At the end of the culture, the cells on the upper surfaces of the filters were removed by wiping with a cotton swab. The filters were fixed in ethanol and were stained with Giemsa. The number of cells exuding to the lower surface was counted in five independent visual fields under a microscope at × 200 (Albini et al, 1987).

### RESULTS

### Initial adhesion step of HepG2 cells to endothelial cells

HepG2 cells strongly adhered to rIL-1-activated HUVECs (Figure 1). The contribution of cell adhesion molecules to the adhesion of HepG2 cells was evaluated using specific monoclonal antibodies. When the rIL-1β-activated HUVECs were pretreated with antibodies directed to E-selectin, ICAM-1 or VCAM-1, adhesion was significantly inhibited only by the treatment with anti-E-selectin antibody (Figure 1A). The effect of anti-ICAM-1 and anti-VCAM-1 antibodies was negligible. When HepG2 cells were treated with anti-carbohydrate antibodies before adhesion to HUVECs, adhesion of the cells was completely abrogated by pretreating the cells with anti-sialyl Lewis X (Figure 1B). These results indicate that the sialyl Lewis X/E-selectin cell adhesion system plays a primary role in the initial adhesion step of HepG2 cells to HUVECs.

© Cancer Research Campaign 1997 

**Figure 4** Effect of concentration (**A**) and incubation time (**B**) on the enhancement of integrin expression on HepG2 cells induced by the rHGF treatment. (**A**) Dose dependency of $\alpha_2$ (●) and $\beta_1$ (○) integrin subunit expression detected by CELISA using anti-CD49b and anti-CD29 antibodies. HepG2 cells were cultured in DMEM containing varying concentrations of rHGF for 24 h. (**B**) Time course of $\alpha_2$ (●) and $\beta_1$ (○) integrin subunit expression. HepG2 cells were incubated in DMEM containing 20 ng ml$^{-1}$ rHGF for 0, 10, 30 min or 1, 2, 4, 8 and 24 h. Each point represents the mean absorbance above background of triplicate assays

### Increased expression of integrins by prolonged culture of HepG2 cells and effect of cytokines

To investigate any possible change in the integrin expression following the first step of adhesion, HepG2 cells were co-cultured with HUVECs for 24 h and subjected to flow cytometric analysis. The results demonstrated that the expression of $\alpha_2\beta_1$ integrin on HepG2 cells was significantly increased by co-cultivation with HUVECs (Figure 2A).

Next, HepG2 cells were treated with various cytokines that are reported to be produced by endothelial cells, to determine which cytokine produced by HUVECs was responsible for the observed up-regulation of $\alpha_2\beta_1$ integrin expression. The results of CELISA are shown in Figure 3. Among the tested cytokines, rHGF had the strongest up-regulating effect on the $\alpha_2\beta_1$ integrin expression. Some other cytokines, such as bFGF or IL-1$\beta$, enhanced the expression, but only weakly. No significant change was noted by the treatment with IL-8, MIP-1$\beta$ or KGF.

The enhancing effect of rHGF was confirmed by flow cytometric analysis. After HepG2 cells were cultured in the presence of 20 ng ml$^{-1}$ rHGF for 24 h, the expression of $\alpha_2\beta_1$ integrin on HepG2 cells was up-regulated to the same level as that obtained by the co-cultivation with HUVECs (Figure 2B).

**Figure 5** The expression of various integrins on HepG2 cells measured by CELISA after 24 h incubation with (■) or without (□) 20 ng ml$^{-1}$ rHGF. Each absorbance represents the mean value above background of duplicate determinations

### Characterization of HGF action on the integrin expression of HepG2 cells

The dose–response of $\alpha_2\beta_1$ integrin expression was determined by CELISA after treatment of HepG2 cells with varying concentrations of rHGF for 24 h. Increase in the expression of both integrins was correlated to the concentration of rHGF, with maximal expressions of the antigens being obtained by the treatment at 20 ng ml$^{-1}$ (Figure 4A).

Figure 4B shows the time course of rHGF action on integrin expression, indicating that an increase in integrin expression was already detectable after 1 h, with an apparent maximum being observed after 24 h.

HepG2 cells express the $\alpha_2$, $\alpha_6$, $\beta_1$ and $\beta_4$ integrin subunits, but the $\alpha_1$, $\alpha_3$, $\alpha_4$, $\alpha_5$, $\beta_2$ and $\beta_3$ integrin chains were not detected in significant amounts. We investigated the expression of various integrin subunits on HepG2 cells by CELISA. Increase of expression was most prominent for the $\beta_1$ integrin subunit (CD29), followed by the $\alpha_2$ subunit (CD49b) after treatment with 20 ng ml$^{-1}$ rHGF for 24 h. No significant change was observed in expression of the other integrins (Figure 5).

**Table 1** Effect of anti-HGF neutralizing antibody on the enhancement of $\alpha_2\beta_1$ integrin expression induced by the addition of rHGF or by the co-cultivation with HUVECs in HepG2 cells

| Treatment of HepG2 cells | Net increase in mean fluorescence intensity (%) |
|---|---|
| Experiment 1 | |
| Addition of rHGF (20 ng ml$^{-1}$) | 100.0 |
| Addition of rHGF (20 ng ml$^{-1}$) +anti-HGF | 0.6 |
| Experiment 2 | |
| Co-cultivation with HUVECs | 100.0 |
| Co-cultivation with HUVECs +anti-HGF | 52.6 |
| Co-cultivation with HUVECs +anti-bFGF | 99.5 |
| Co-cultivation with HUVECs +anti-IL-1$\beta$ | 96.5 |

In experiment 1, the net increase in mean fluorescence intensity of $\alpha_2$-integrin (CD49b) on HepG2 cells that had been treated with 20 ng ml$^{-1}$ (see Figure 2B), compared with that of non-treated HepG2 cells, was taken as 100%. In experiment 2, the net increase in mean fluorescence intensity of $\alpha_2$-integrin on HepG2 cells that had been co-cultured with HUVECs (see Figure 2A), was taken as 100%.

 © Cancer Research Campaign 1997

**Figure 6** Effects of rHGF on the attachment of the HepG2 cells to collagen I and laminin. In **A**, HepG2 cells were cultured in the presence (■) or absence (□) of 20 ng ml$^{-1}$ rHGF for 24 h and allowed to attach for 30 min to wells coated with collagen I or laminin. Bars indicate s.d. Statistical significance was tested by Student's *t*-test. In **B**, HepG2 cells, which had been cultured with (■) or without (□) 20 ng ml$^{-1}$ rHGF for 24 h, were allowed to attach for 30 min to the collagen-coated wells in the presence of blocking antibodies directed to CD49b ($\alpha_2$-integrin), CD49f ($\alpha_6$-integrin) or CD29 ($\beta_1$-integrin). Bars indicate s.d.

### Effect of anti-HGF antibody on integrin expression of HepG2 cells

An inhibition experiment exploying neutralizing antisera against rHGF was performed to determine whether the enhancement of the integrin expression by HUVECs is mediated by HGF produced by HUVECs. This rabbit antisera preparation completely inhibited the up-regulation induced by rHGF. On the other hand, HUVEC-induced enhancement of the $\alpha_2\beta_1$ integrin expression was inhibited by about 50%.

This result indicates that at least 50% of the enhancing effect on the integrin expression, which was exerted by the co-cultivation with HUVECs, is mediated by HGF produced by HUVECs. The mechanisms involved in the other 50% remain unclear. One possible explanation for the latter would be the additional action of other cytokines that have a weak enhancing effect, such as bFGF and/or IL-1β. However, the addition of rabbit anti-IL-1β or anti-bFGF antibody did not abolish the enhancement of the integrin expression (Table 1). Another possibility would be that the neutralization of rHGF is difficult when the stimulative effect is conveyed by direct physical contact between HUVECs and HepG2 cells.

### Enhancement of adhesion to collagen and of transmigratory activity

The attachment assay to collagen I and laminin of HepG2 cells was carried out to investigate whether the increased expression of $\alpha_2\beta_1$ integrin really affects the adhesive behaviour of the HepG2 cells to the putative ECM ligands for the integrin. As shown in Figure 6A, HepG2 cells cultured in the presence of rHGF showed an increased binding activity to collagen, the putative ligand for the $\alpha_2\beta_1$ integrin. On the other hand, the attachment to laminin showed no significant change. This result is in line with the finding that the expression of the $\alpha_6$ integrin subunit on HepG2 cells showed no change upon stimulation with rHGF, since laminin is the putative ligand for the $\alpha_6\beta_1$ integrin.

The adhesion of HGF-treated HepG2 cells to collagen was nearly completely inhibited by the treatment with anti-$\alpha_2$ or anti-$\beta_1$ antibodies, but not with anti-$\alpha_6$ antibody (Figure 6B). This result indicates that the augmentation in the adhesion was caused by the activation of $\alpha_2\beta_1$-integrin by rHGF.

We also performed chemoinvasion experiments to evaluate the effect of the co-cultivation with HUVECs or of the addition of rHGF on the transmigratory activity of the HepG2 cells (Figure 7). In these experiments, the lower wells of the 24-well plates contained collagen, and the cancer cells migrating through Matrigel and appearing at the lower membrane surface were evaluated. HepG2 cells co-cultured with HUVECs had a higher invading activity than non-treated HepG2 cells (Figure 7A). The same level of increment in the transmigratory activity was attained when HepG2 cells were stimulated with rHGF (Figure 7B).

### DISCUSSION

Exudation of leucocytes to endothelial cells is known to be initiated by the cell adhesion mediated by selectins and carbohydrate ligands (Stoolman, 1989; Springer and Lasky, 1991). This is followed by the second step of cell adhesion that is mediated by integrins and corresponding molecules of the immunoglobulin superfamily, such as ICAM-1 and VCAM-1, with this step assumed to induce the exudation of leucocytes into extravascular tissues (Stoolman, 1989; Springer and Lasky, 1991). LFA-1, the ligand for ICAM-1, and VLA-4, the ligand for VCAM-1, are abundantly expressed on leucocytes. In contrast to this, expression of LFA-1 or VLA-4 is relatively rare in epithelial cancer cells, while $\alpha_2$, $\alpha_3$, $\alpha_6$, $\beta_1$ and $\beta_4$ integrin subunits are commonly expressed on most cancer cells (Weinel et al, 1992; Albelda, 1993; Volpes et al, 1993; our unpublished results).

In this study, HepG2 cells did not express LFA-1 or VLA-4, similar to many other epithelial cancer cells. The integrins expressed significantly on HepG2 cells were $\alpha_2$, $\alpha_6$, $\beta_1$ and $\beta_4$ integrin chains. Following the first step of adhesion to endothelial cells mediated by E-selectin and sialyl Lewis X, only the expression of $\alpha_2\beta_1$ integrin was enhanced on HepG2 cells by co-cultivation with HUVECs. This effect of HUVECs was mimicked by the addition of rHGF to the culture medium. A significant portion of the enhancing effect was identified to be owing to the action of HGF produced by endothelial cells, by the experiments using neutralizing antibody. MIP-1β and IL-8 are suggested to be involved in the activation of leucocyte integrins in leucocyte–endothelial adhesion (Kuijpers et al, 1992; Tanaka et al, 1993). In the case of HepG2 cells, MIP-1β and IL-8 were devoid of any detectable effect on integrin expression. MIP-1β and IL-8

**Figure 7** Effect of rHGF or HUVECs on the transmigratory activity of HepG2 cells. The experimental procedures are shown in the insets of the figure. (**A**) Transmigratory activity of HepG2 cells ($7 \times 10^4$) in the presence (■) or absence (□) of HUVECs. (**B**) Transmigratory activity of HepG2 cells in the presence (■) or absence (□) of 20 ng $ml^{-1}$ rHGF. The cells appearing on the lower surface of the filters were stained with Giemsa, and the number of cells was counted in five visual fields under a microscope (× 200). Each result represents the median value above the number of cells exuded when collagen was omitted are described as acting primarily on the leucocytes, and little literature reporting their action on epithelial cells is available.

Hepatocyte growth factor (HGF) was first identified as a potent stimulator of hepatocyte growth and DNA synthesis (Matsumoto and Nakamura, 1992). Following its purification and sequencing, its identity with scatter factor was established (Nakamura et al, 1989). It is well established that HGF is produced by endothelial cells as well as fibroblasts and macrophages (Noji et al, 1990; Matsumoto et al, 1992). HGF produced by the endothelial cells was even suggested to affect the adhesive activity of lymphocytes at the vessel wall (Adams et al, 1994). Various inflammatory cytokines, including IL-1β, are known to stimulate the production of HGF (Matsumoto et al, 1992). In this context, it is notable that the expression of E-selectin, which plays an important role as a ligand for carbohydrate antigens in the adhesion to cancer cells, is also induced by inflammatory cytokines, such as IL-1β (Bevilacqua et al, 1989). Taken together, it is surmised that production of HGF is enhanced at the vascular endothelium that expresses E-selectin. The possibility that cancer cells, such as HepG2, eventually produce HGF in response to some stimuli exerted by activated endothelial cells remains.

In line with the increase in the $\alpha_2\beta_1$ integrin expression, the attachment of HepG2 cells to collagen was enhanced by the addition of rHGF. We also carried out a chemoinvasion assay using an experimental system that resembles the in vivo condition of cancer cell transmigration at the vessel wall. When collagen was used as the chemoattractant, the invasion of HepG2 cells was greatly enhanced by co-cultivation with HUVEC monolayers. A similar level of enhancement in transmigratory activity was observed by the addition of rHGF to HepG2 cells. These findings suggest that HGF facilitates exudation of HepG2 cells into extravascular tissues. HGF is known to enhance cell mobility (Tajima et al, 1992; Shibamoto et al, 1992), and a part of this activity would be related to its effect on integrin expression as described in this study.

Recent evidence suggests that HGF increases the motility and invasiveness of cancer cells both under in vitro and in vivo conditions (Tajima et al, 1992; Shibamoto et al, 1992; Yoshinaga et al, 1993; Yamashita et al, 1994). In this study, we propose that HGF would also be involved in the haematogenous metastasis of cancer, at the step of adhesion of cancer cells to endothelial cells. HGF has been demonstrated as displaying a variety of biological activities on various target cell types, including epithelial neoplasma (Tajima et al, 1992; Shibamoto et al, 1992; Yoshinaga et al, 1993). The wide spectrum of targets of HGF corresponds to the expression of its receptor, a tyrosine kinase, first described as the c-*met* proto-oncogene product. Several investigators reported that amplification of the c-*met* proto-oncogene may participate in carcinogenesis and progression of gastric cancer (Kuniyasu et al, 1992), colorectal cancer (Liu et al, 1992) and thyroid cancer (Di Renzo et al, 1992). This would also imply that the HGF-mediated enhancement of integrin expression would be limited to the cancer cells that express the c-*met* oncogene product. In our hands, HGF exerted a similar effect on some cancer cells, such as A431 cells, besides HepG2, but had no effect on some other cancer cells. For such cancer cells that do not respond to HGF, other cytokines, such as KGF, heparin-binding EGF (HB-EGF) or amphiregulin, can be considered as candidates for the cytokines that act in the second step of adhesion. These cytokines share characteristics common to HGF in that their main target is epithelial cells and they are produced by endothelial cells and associated with proteoglycan-like structures at the endothelial cell surface. Our preliminary results indicated that HB-EGF had an enhancing effect on integrin expression on some oesophageal and breast cancer cells (T Narita, N Kawakami and R Kannagi, manuscript in preparation). The cell lineage restriction in the action of HGF and other cytokines in the second step of cell adhesion is in contrast to the situation in the first step of adhesion, in which the selectin-mediated adhesion of cancer cells to endothelial cells is commonly observed in a wide range of epithelial cancer cells.

### ABBREVIATIONS

Abbreviations E-selectin (ELAM-1), endothelial–leucocyte adhesion molecule 1; ICAM-1, intercellular adhesion molecule 1; VCAM-1, vascular cell adhesion molecule 1; LFA-1, lymphocyte function-associated antigen 1; VLA-4, very-late antigen 4; HUVEC, human umbilical vein endothelial cell; IL-1, interleukin 1.

### ACKNOWLEDGEMENTS

This work was supported in part by a grant-in-aid for the second term comprehensive ten-year strategy for cancer control from the Ministry of Health and Welfare, Japan, and grants-in-aid for scientific research from the Ministry of Education, Science and Culture, Japan (07672510, 07671349 and 08672659).

### REFERENCES

Adams DH, Harvath L, Bottaro DP, Interrante R, Catalano G, Tanaka Y, Strain A, Hubscher SG and Shaws (1994) Hepatocyte growth factor and macrophage inflammatory protein 1-β: structurally distinct cytokines that induce rapid cytoskeletal changes and subset-preferential migration in T cells. *Proc Natl Acad Sci USA* **91**: 7144–7148

Albelda SM (1993) Role of integrins and other cell adhesion molecules in tumor progression and metastasis. *Lab Invest* **68**: 4–17

© Cancer Research Campaign 1997

Albini A, Iwamoto Y, Kleinman HK, Martin GR, Aaronson S A, Kozlowski JM and Mcewan RN (1987) A rapid *in vitro* assay for quantitating the invasive potential of tumor cells. *Cancer Res* **47**: 3239–3245

Alexandroff AB, Jackson AM, Esuvaranathan K, Prescott S and James K (1994) Autocrine regulation of ICAM-1 expression on bladder cancer cell lines: evidence for the role of IL-1$\alpha$. *Immunol Lett* **40**: 117–124

Banks RE, Gearing AJH, Hemingway IK, Norfolk DR, Perren TJ and Selby PJ (1993) Circulating intercellular adhesion molecule-1 (ICAM-1), E-selectin and vascular cell adhesion molecule-1 (VCAM-1) in human malignancies. *Br. J Cancer* **68**: 122–124.

Bevilacqua MP, Pober JS, Mendrick DL, Cotran RS and Gimbrone MAJ (1987) Identification of an inducible endothelial–leukocyte adhesion molecule. *Proc Natl Acad Sci USA* **84**: 9238–9242

Bevilacqua MP, Stengelin S, Gimbrone MAJ and Seed B (1989) Endothelial leukocyte adhesion molecule 1: an inducible receptor for neutrophils related to complement regulatory proteins and lectins. *Science* **243**: 1160–1165

Carter WG, Wayner EA, Bouchard TS and Kaur P (1990) The role of integrins $\alpha_2\beta_1$ and $\alpha_3\beta_1$ in cell–cell and cell–substrate adhesion of human epidermal cells. *J Cell Biol* **110**: 1387–1404

Dejana E, Martin Padura I, Lauri D, Bernasconi S, Bani MR, Garofalo A, Giavazzi R, Magnani J, Mantovani A and Menard S (1992) Endothelial leukocyte adhesion molecule-1-dependent adhesion of colon carcinoma cells to vascular endothelium is inhibited by an antibody to Lewis fucosylated type I carbohydrate chain. *Lab Invest* **66**: 324–330

Di Renzo MF, Olivero M, Ferro S, Prat M, Bongarzone I, Pilotti S, Belfiore A, Costantino A, Vigneri R, Pierotti MA and Comoglio PM (1992) Overexpression of the c-*MET*/HGF receptor gene in human thyroid carcinomas. *Oncogene* **7**: 2549–2553

Giavazzi R, Foppolo M, Dossi R and Remuzzi A (1993) Rolling and adhesion of human tumor cells on vascular endothelium under physiological flow conditions. *J Clin Invest* **92**: 3038–3044

Hakomori S (1992) $Le^x$ and related structures as adhesion molecules. *Histochem J* **24**: 771–776

Hayashi O, Akashi M, Fujime M, Hanazawa K and Kitagawa R (1994) Detection of interleukin-1 activity in human bladder cancer cell lines. *J Urol* **151**: 750–753

Kuijpers TW, Hakkert BC, Hart MH and Roos D (1992) Neutrophil migration across monolayers of cytokine-prestimulated endothelial cells: a role for platelet-activating factor and IL-8. *J Cell Biol* **117**: 565–572

Kuniyasu H, Yasui W, Kitadai Y, Yokozaki H, Ito H and Tahara E (1992) Frequent amplification of the *c-met* gene in scirrhous type stomach cancer. *Biochem Biophys Res Commun* **189**: 227–232

Li By, Mohanraj D, Olson MC, Moradi M, Twiggs L, Carson LF and Ramakrishnan S (1992) Human ovarian epithelial cancer cells cultures *in vitro* express both interleukin 1$\alpha$ and $\beta$ genes. *Cancer Res* **52**: 2248–2252

Liu C, Park M and Tsao MS (1992) Overexpression of c-*met* proto-oncogene but not epidermal growth factor receptor or c-*erb*B-2 in primary human colorectal carcinomas. *Oncogene* **7**: 181–185

Lowe JB, Stoolman LM, Nair RP, Larsen RD, Berhend TL and Marks RM (1990) ELAM-1-dependent cell adhesion to vascular endothelium determined by a transfected human fucosyltransferase cDNA. *Cell* **63**: 475–484

Majuri M-L, Mattila P and Renkonen R (1992) Recombinant E-selectin-protein mediates tumor cell adhesion via sialyl-$Le^a$ and sialyl-$Le^x$. *Biochem Biophys Res Commun* **182**: 1376–1382

Matsumoto K and Nakamura T (1992) Hepatocyte growth factor: molecular structure, roles in liver regeneration, and other biological functions. *Crit Rev Oncogen* **3**: 27–54

Matsumoto K, Okazaki H and Nakamura T (1992) Up-regulation of hepatocyte growth factor gene expression by interleukin-1 in human skin fibroblasts. *Biochem Biophys Res Commun* **188**: 235–243

Merwin JR, Madri JA and Lynch M (1992) Cancer cell binding to E-selectin transfected human endothelia. *Biochem Biophys Res Commun* **189**: 315–323

Montesano R, Matsumoto K, Nakamura T and Orci L (1991) Identification of a fibroblast-derived epithelial morphogen as hepatocyte growth factor. *Cell* **67**: 901–908

Nakamura T, Nishizawa T, Hagiya M, Seki T, Shimonishi M, Sugimura A, Tashiro K and Shimizu S (1989) Molecular cloning and expression of human hepatocyte growth factor. *Nature* **342**: 440–443

Noji S, Tashiro K, Koyama E, Nohno T, Ohyama K, Taniguchi S and Nakamura T (1990) Expression of hepatocyte growth factor gene in endothelial and Kupffer cells of damaged rat livers, as revealed by *in situ* hybridization. *Biochem Biophys Res Commun* **173**: 42–47

Ohmori K, Takada A, Ohwaki I, Takahashi N, Furukawa Y, Maeda M, Kiso M, Hasegawa A, Kannagi M and Kannagi R (1993) A distinct type of sialyl Lewis X antigen defined by a novel monoclonal antibody is selectively expressed on helper memory T cells. *Blood* **82**: 2797–2805

Phillips ML, Nudelman E, Gaeta FCA, Perez M, Singhal AK, Hakomori S and Paulson JC (1990) ELAM-1 mediates cell adhesion by recognition of a carbohydrate ligand, sialyl-$Le^x$. *Science* **250**: 1130–1132

Shibamoto S, Hayakawa M, Hori T, Oku N, Miyazawa K, Kitamura N and Ito F (1992) Hepatocyte growth factor and transforming growth factor- $\beta$ stimulate both cell growth and migration of human gastric adenocarcinoma cells. *Cell Struct Funct* **17**: 185–190

Springer TA and Lasky LA (1991) Sticky sugars for selectins. *Nature* **349**: 196–197

Staatz WD, Rajpara SM, Wayner EA, Carter WG and Santoro SA (1989) The membrane glycoprotein Ia–IIa (VLA-2) complex mediates the $Mg^{++}$-dependent adhesion of platelets to collagen. *J Cell Biol* **108**: 1917–1924

Stoolman LM (1989) Adhesion molecules controlling lymphocyte migration. *Cell* **56**: 907–910

Tajima H, Matsumoto K and Nakamura T (1992) Regulation of cell growth and motility by hepatocyte growth factor and receptor expression in various cell species. *Exp Cell Res* **202**: 423–431

Takada A, Ohmori K, Takahashi N, Tsuyuoka K, Yago K, Zenita K, Hasegawa A and Kannagi R (1991) Adhesion of human cancer cells to vascular endothelium mediated by a carbohydrate antigen, sialyl Lewis A. *Biochem Biophys Res Commun* **179**: 713–719

Takada A, Ohmori K, Yoneda T, Tsuyuoka K, Hasegawa A, Kiso M and Kannagi R (1993) Contribution of carbohydrate antigens sialyl Lewis A and sialyl Lewis X to adhesion of human cancer cells to vascular endothelium. *Cancer Res* **53**: 354–361

Tanaka Y, Adams DH, Hubscher S, Hirano H, Siebenlist U and Shaw S (1993) T-cell adhesion induced by proteoglycan-immobilized cytokine MIP-1$\beta$. *Nature* **361**: 79–82

Volpes R, Van Den Oord JJ and Desmet VJ (1993) Integrins as differential cell lineage markers of primary liver tumors. *Am J Pathol* **142**: 1483–1492

Weinel RJ, Rosendahl A, Neumann K, Chaloupka B, Erb D, Rothmund M and Santoso S (1992) Expression and function of VLA-$\alpha_2$-$\alpha_3$, –$\alpha_5$ and –$\alpha_6$-integrin receptors in pancreatic carcinoma. *Int J Cancer* **52**: 827–833

Yamashita J, Ogawa M, Yamashita S, Nomura K, Kuramoto M, Saishoji T and Shin S (1994) Immunoreactive hepatocyte growth factor is a strong and independent predictor of recurrence and survival in human breast cancer. *Cancer Res* **54**: 1630–1633

Ye C, Kiriyama K, Mitsuoka C, Kannagi R, Ito K, Watanabe T, Kondo K, Akiyama S and Takagi H (1995) Expression of E-selection on endothelial cells of small veins and proliferating vessels in human colorectal carcinoma. *Int J Cancer* **61**: 455–460

Yoshinaga Y, Matsuno Y, Fujita S, Nakamura T, Kikuchi M, Shimosato Y and Hirohashi S (1993) Immunohistochemical detection of hepatocyte growth factor/scatter factor in human cancerous and inflammatory lesions of various organs. *Jpn J Cancer Res* **84**: 1150–1158

© Cancer Research Campaign 1997


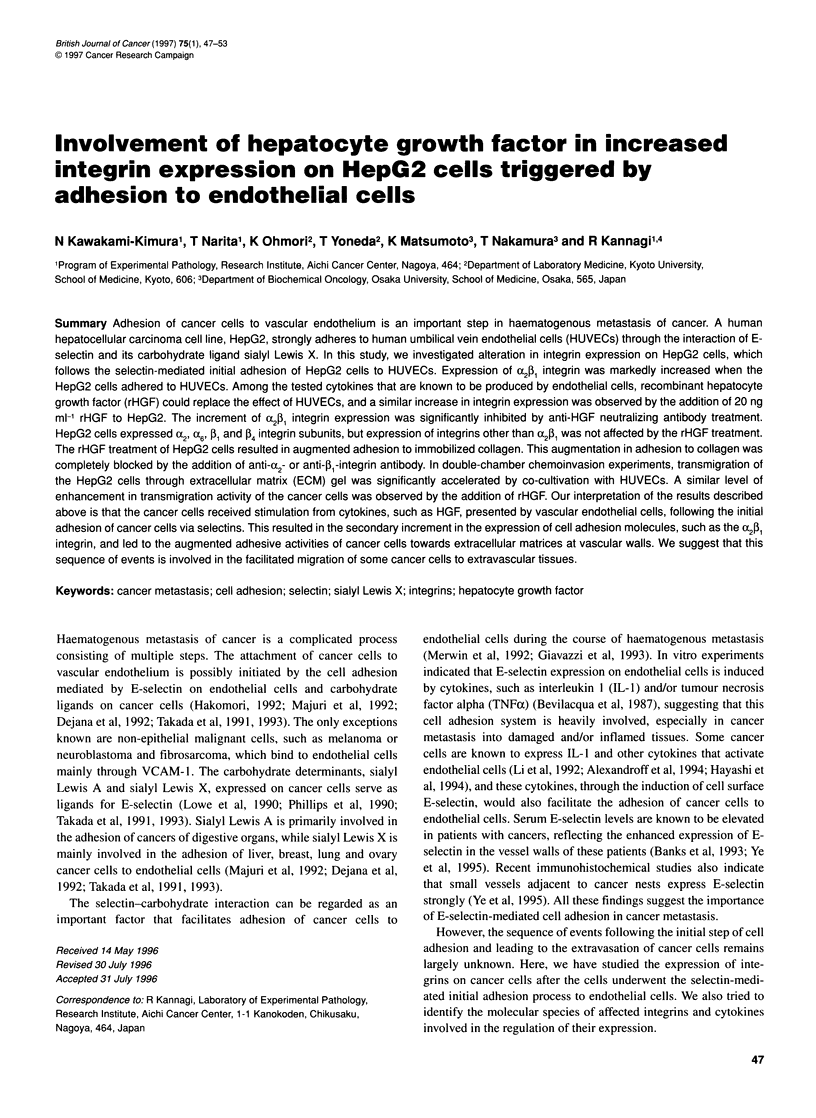

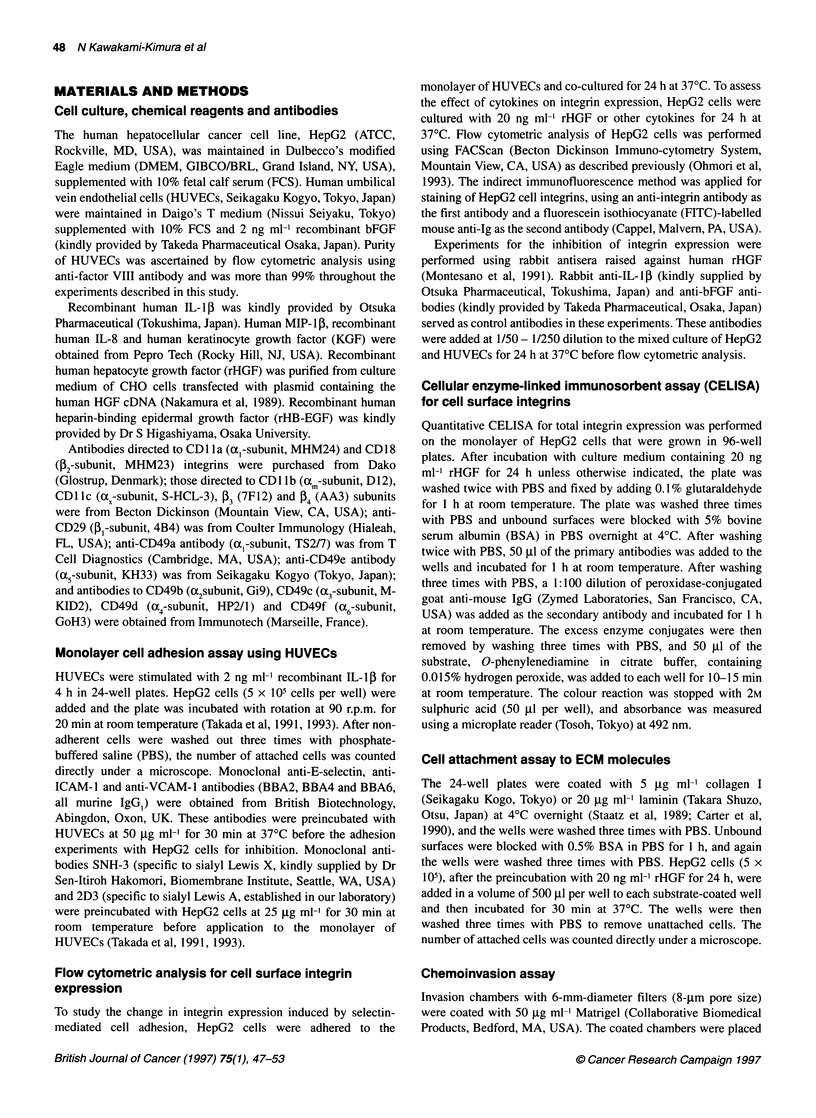

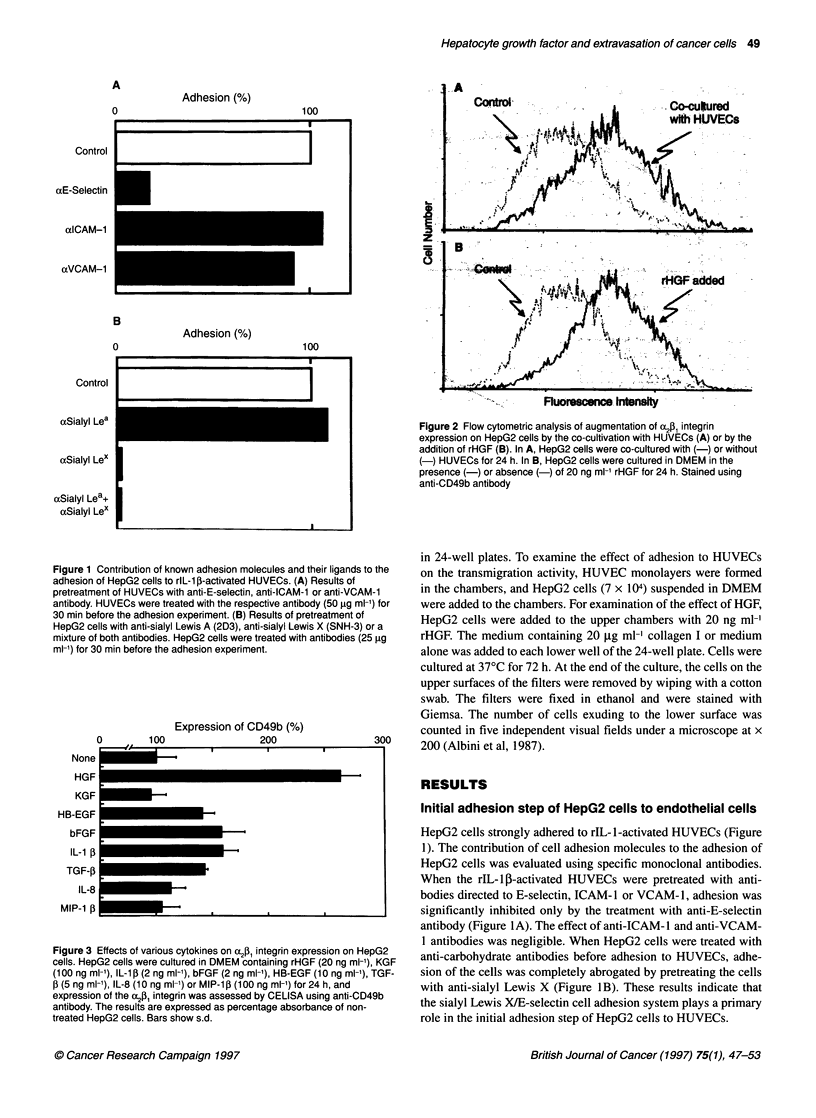

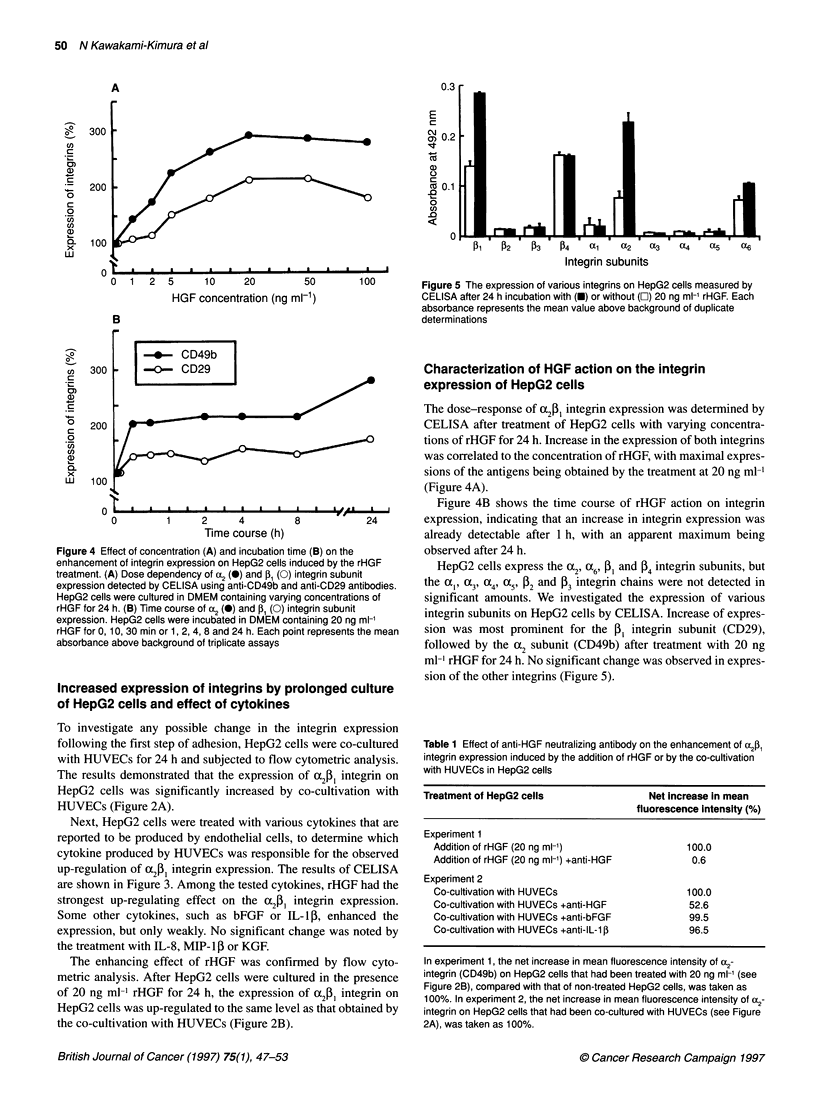

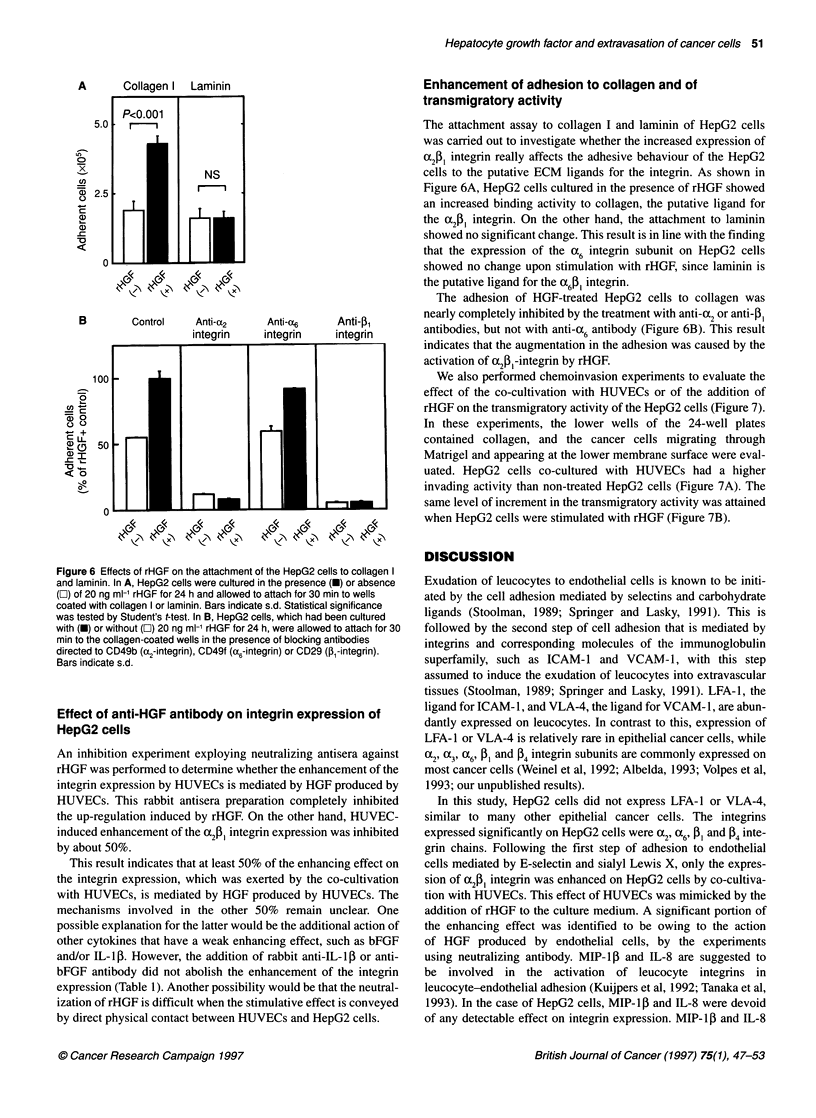

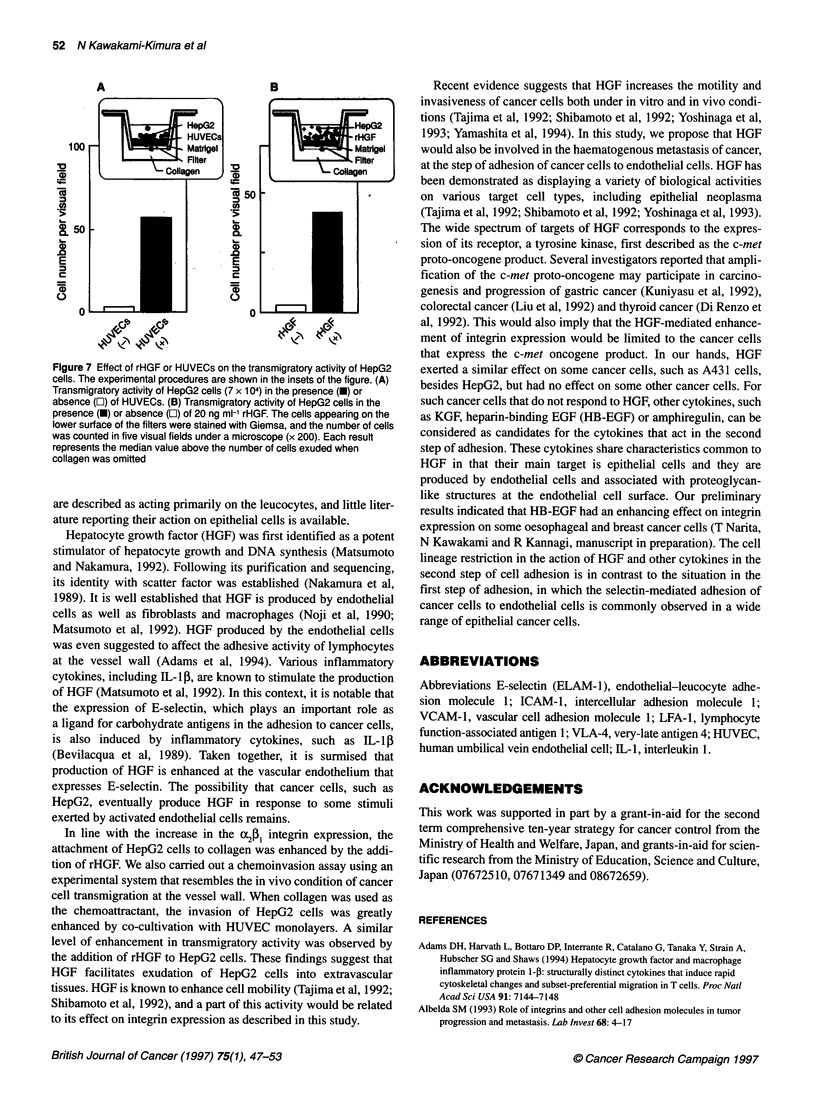

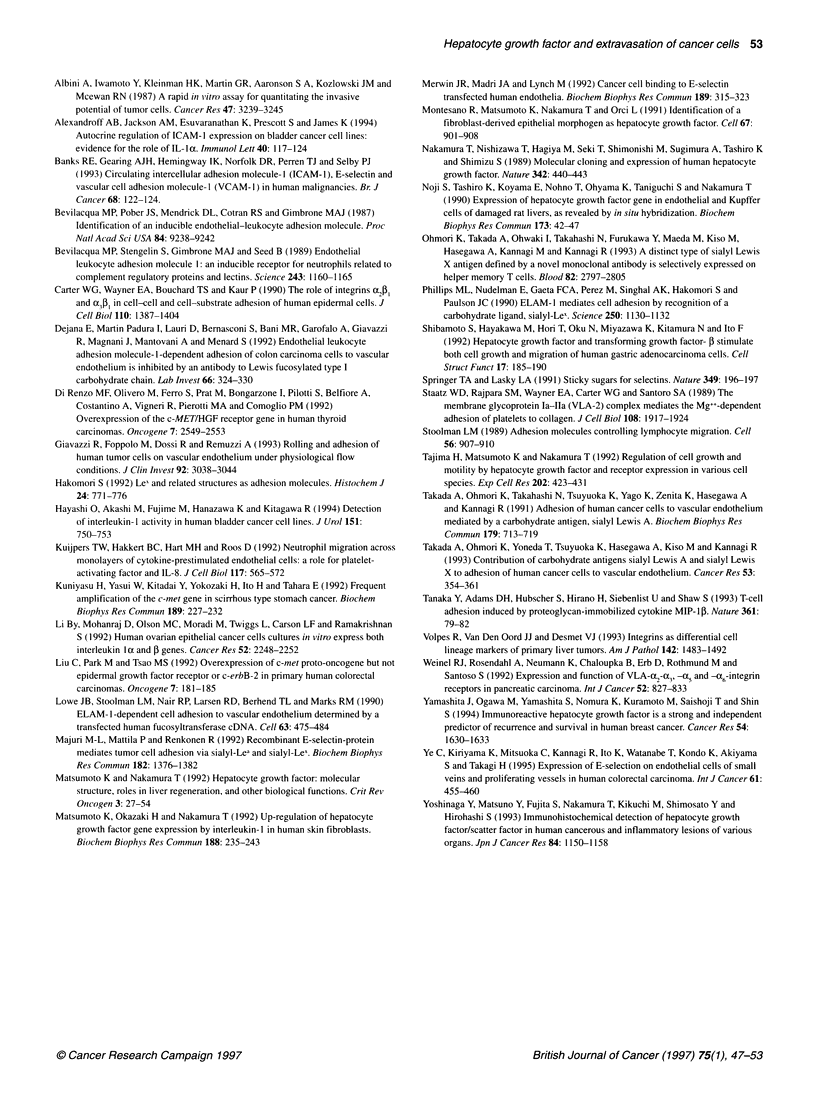


## References

[OCR_00839] Adams D. H., Harvath L., Bottaro D. P., Interrante R., Catalano G., Tanaka Y., Strain A., Hubscher S. G., Shaw S. (1994). Hepatocyte growth factor and macrophage inflammatory protein 1 beta: structurally distinct cytokines that induce rapid cytoskeletal changes and subset-preferential migration in T cells.. Proc Natl Acad Sci U S A.

[OCR_00847] Albelda S. M. (1993). Role of integrins and other cell adhesion molecules in tumor progression and metastasis.. Lab Invest.

[OCR_00857] Albini A., Iwamoto Y., Kleinman H. K., Martin G. R., Aaronson S. A., Kozlowski J. M., McEwan R. N. (1987). A rapid in vitro assay for quantitating the invasive potential of tumor cells.. Cancer Res.

[OCR_00860] Alexandroff A. B., Jackson A. M., Esuvaranathan K., Prescott S., James K. (1994). Autocrine regulation of ICAM-1 expression on bladder cancer cell lines: evidence for the role of IL-1 alpha.. Immunol Lett.

[OCR_00865] Banks R. E., Gearing A. J., Hemingway I. K., Norfolk D. R., Perren T. J., Selby P. J. (1993). Circulating intercellular adhesion molecule-1 (ICAM-1), E-selectin and vascular cell adhesion molecule-1 (VCAM-1) in human malignancies.. Br J Cancer.

[OCR_00871] Bevilacqua M. P., Pober J. S., Mendrick D. L., Cotran R. S., Gimbrone M. A. (1987). Identification of an inducible endothelial-leukocyte adhesion molecule.. Proc Natl Acad Sci U S A.

[OCR_00876] Bevilacqua M. P., Stengelin S., Gimbrone M. A., Seed B. (1989). Endothelial leukocyte adhesion molecule 1: an inducible receptor for neutrophils related to complement regulatory proteins and lectins.. Science.

[OCR_00881] Carter W. G., Wayner E. A., Bouchard T. S., Kaur P. (1990). The role of integrins alpha 2 beta 1 and alpha 3 beta 1 in cell-cell and cell-substrate adhesion of human epidermal cells.. J Cell Biol.

[OCR_00886] Dejana E., Martin-Padura I., Lauri D., Bernasconi S., Bani M. R., Garofalo A., Giavazzi R., Magnani J., Mantovani A., Menard S. (1992). Endothelial leukocyte adhesion molecule-1-dependent adhesion of colon carcinoma cells to vascular endothelium is inhibited by an antibody to Lewis fucosylated type I carbohydrate chain.. Lab Invest.

[OCR_00894] Di Renzo M. F., Olivero M., Ferro S., Prat M., Bongarzone I., Pilotti S., Belfiore A., Costantino A., Vigneri R., Pierotti M. A. (1992). Overexpression of the c-MET/HGF receptor gene in human thyroid carcinomas.. Oncogene.

[OCR_00901] Giavazzi R., Foppolo M., Dossi R., Remuzzi A. (1993). Rolling and adhesion of human tumor cells on vascular endothelium under physiological flow conditions.. J Clin Invest.

[OCR_00906] Hakomori S. (1992). Le(X) and related structures as adhesion molecules.. Histochem J.

[OCR_00910] Hayashi O., Akashi M., Fujime M., Hanazawa K., Kitagawa R. (1994). Detection of interleukin-1 activity in human bladder cancer cell lines.. J Urol.

[OCR_00915] Kuijpers T. W., Hakkert B. C., Hart M. H., Roos D. (1992). Neutrophil migration across monolayers of cytokine-prestimulated endothelial cells: a role for platelet-activating factor and IL-8.. J Cell Biol.

[OCR_00920] Kuniyasu H., Yasui W., Kitadai Y., Yokozaki H., Ito H., Tahara E. (1992). Frequent amplification of the c-met gene in scirrhous type stomach cancer.. Biochem Biophys Res Commun.

[OCR_00925] Li B. Y., Mohanraj D., Olson M. C., Moradi M., Twiggs L., Carson L. F., Ramakrishnan S. (1992). Human ovarian epithelial cancer cells cultures in vitro express both interleukin 1 alpha and beta genes.. Cancer Res.

[OCR_00930] Liu C., Park M., Tsao M. S. (1992). Overexpression of c-met proto-oncogene but not epidermal growth factor receptor or c-erbB-2 in primary human colorectal carcinomas.. Oncogene.

[OCR_00935] Lowe J. B., Stoolman L. M., Nair R. P., Larsen R. D., Berhend T. L., Marks R. M. (1990). ELAM-1--dependent cell adhesion to vascular endothelium determined by a transfected human fucosyltransferase cDNA.. Cell.

[OCR_00940] Majuri M. L., Mattila P., Renkonen R. (1992). Recombinant E-selectin-protein mediates tumor cell adhesion via sialyl-Le(a) and sialyl-Le(x).. Biochem Biophys Res Commun.

[OCR_00945] Matsumoto K., Nakamura T. (1992). Hepatocyte growth factor: molecular structure, roles in liver regeneration, and other biological functions.. Crit Rev Oncog.

[OCR_00950] Matsumoto K., Okazaki H., Nakamura T. (1992). Up-regulation of hepatocyte growth factor gene expression by interleukin-1 in human skin fibroblasts.. Biochem Biophys Res Commun.

[OCR_00955] Merwin J. R., Madri J. A., Lynch M. (1992). Cancer cell binding to E-selectin transfected human endothelia.. Biochem Biophys Res Commun.

[OCR_00958] Montesano R., Matsumoto K., Nakamura T., Orci L. (1991). Identification of a fibroblast-derived epithelial morphogen as hepatocyte growth factor.. Cell.

[OCR_00963] Nakamura T., Nishizawa T., Hagiya M., Seki T., Shimonishi M., Sugimura A., Tashiro K., Shimizu S. (1989). Molecular cloning and expression of human hepatocyte growth factor.. Nature.

[OCR_00968] Noji S., Tashiro K., Koyama E., Nohno T., Ohyama K., Taniguchi S., Nakamura T. (1990). Expression of hepatocyte growth factor gene in endothelial and Kupffer cells of damaged rat livers, as revealed by in situ hybridization.. Biochem Biophys Res Commun.

[OCR_00974] Ohmori K., Takada A., Ohwaki I., Takahashi N., Furukawa Y., Maeda M., Kiso M., Hasegawa A., Kannagi M., Kannagi R. (1993). A distinct type of sialyl Lewis X antigen defined by a novel monoclonal antibody is selectively expressed on helper memory T cells.. Blood.

[OCR_00980] Phillips M. L., Nudelman E., Gaeta F. C., Perez M., Singhal A. K., Hakomori S., Paulson J. C. (1990). ELAM-1 mediates cell adhesion by recognition of a carbohydrate ligand, sialyl-Lex.. Science.

[OCR_00985] Shibamoto S., Hayakawa M., Hori T., Oku N., Miyazawa K., Kitamura N., Ito F. (1992). Hepatocyte growth factor and transforming growth factor-beta stimulate both cell growth and migration of human gastric adenocarcinoma cells.. Cell Struct Funct.

[OCR_00991] Springer T. A., Lasky L. A. (1991). Cell adhesion. Sticky sugars for selectins.. Nature.

[OCR_00992] Staatz W. D., Rajpara S. M., Wayner E. A., Carter W. G., Santoro S. A. (1989). The membrane glycoprotein Ia-IIa (VLA-2) complex mediates the Mg++-dependent adhesion of platelets to collagen.. J Cell Biol.

[OCR_00997] Stoolman L. M. (1989). Adhesion molecules controlling lymphocyte migration.. Cell.

[OCR_01001] Tajima H., Matsumoto K., Nakamura T. (1992). Regulation of cell growth and motility by hepatocyte growth factor and receptor expression in various cell species.. Exp Cell Res.

[OCR_01006] Takada A., Ohmori K., Takahashi N., Tsuyuoka K., Yago A., Zenita K., Hasegawa A., Kannagi R. (1991). Adhesion of human cancer cells to vascular endothelium mediated by a carbohydrate antigen, sialyl Lewis A.. Biochem Biophys Res Commun.

[OCR_01012] Takada A., Ohmori K., Yoneda T., Tsuyuoka K., Hasegawa A., Kiso M., Kannagi R. (1993). Contribution of carbohydrate antigens sialyl Lewis A and sialyl Lewis X to adhesion of human cancer cells to vascular endothelium.. Cancer Res.

[OCR_01018] Tanaka Y., Adams D. H., Hubscher S., Hirano H., Siebenlist U., Shaw S. (1993). T-cell adhesion induced by proteoglycan-immobilized cytokine MIP-1 beta.. Nature.

[OCR_01023] Volpes R., van den Oord J. J., Desmet V. J. (1993). Integrins as differential cell lineage markers of primary liver tumors.. Am J Pathol.

[OCR_01027] Weinel R. J., Rosendahl A., Neumann K., Chaloupka B., Erb D., Rothmund M., Santoso S. (1992). Expression and function of VLA-alpha 2, -alpha 3, -alpha 5 and -alpha 6-integrin receptors in pancreatic carcinoma.. Int J Cancer.

[OCR_01032] Yamashita J., Ogawa M., Yamashita S., Nomura K., Kuramoto M., Saishoji T., Shin S. (1994). Immunoreactive hepatocyte growth factor is a strong and independent predictor of recurrence and survival in human breast cancer.. Cancer Res.

[OCR_01038] Ye C., Kiriyama K., Mistuoka C., Kannagi R., Ito K., Watanabe T., Kondo K., Akiyama S., Takagi H. (1995). Expression of E-selectin on endothelial cells of small veins in human colorectal cancer.. Int J Cancer.

[OCR_01045] Yoshinaga Y., Matsuno Y., Fujita S., Nakamura T., Kikuchi M., Shimosato Y., Hirohashi S. (1993). Immunohistochemical detection of hepatocyte growth factor/scatter factor in human cancerous and inflammatory lesions of various organs.. Jpn J Cancer Res.

